# Draft genome sequence of *Enterobacter roggenkampii* strain B1-01, a potential plant-growth promoting bacterium isolated from the roots of bamboo

**DOI:** 10.1128/mra.00707-25

**Published:** 2025-10-29

**Authors:** Camille Andrea R. Flores, Paul Christian T. Gloria, Maria Auxilia T. Siringan, Mary Ann Cielo V. Relucio-San Diego

**Affiliations:** 1 Natural Sciences Research Institute, University of the Philippines, Diliman 54727, Quezon City, Philippines; University of Wisconsin-Madison, Madison, Wisconsin, USA

**Keywords:** PGPB, *Enterobacter roggenkampii*, bamboo roots, WGS

## Abstract

We report the draft genome sequence of *Enterobacter roggenkampii* strain B1-01, isolated from the roots of bamboo in Cagayan, Philippines. The assembled genome is 4.6 Mb in size, with a G + C content of 56.2% and 4,305 protein-coding genes. Genome mining identified genes involved in plant growth promotion and secondary metabolite production.

## ANNOUNCEMENT

Plant growth-promoting bacteria (PGPB) stimulate plant growth and offer protection from pathogens and abiotic stress through various mechanisms (1). Strains of *Enterobacter* spp. have been reported as PGPB, exhibiting beneficial effects on crop plants (2–5). *Enterobacter roggenkampii* strain B1-01 was previously isolated from the roots of common bamboo (*Bambusa vulgaris*) planted in Cagayan, Philippines (17°52′4.81″ N; 121°44′48.54″ E) (6). It exhibited plant growth-promoting (PGP) traits *in vitro*, including phosphate solubilization, indole-3-acetic acid production, and siderophore synthesis (6). The *nifH* gene was also detected through PCR (6). Genome analysis of B1-01 will facilitate the detection of genes involved in its PGP mechanisms.

B1-01 was isolated by inoculating serial dilutions of a surface-sterilized root sample into N-free basal medium (P. Jurtshuk, University of Houston, personal communication, 1988) and incubating at 28°C for 48 h. Subsequently, 0.1 mL aliquots were spread plated in duplicates onto Burk’s N-free medium (HiMedia, India) agar (BMA) plates and incubated under the same conditions. Morphologically distinct colonies were purified by streaking onto fresh BMA (6). Strain B1-01 was then grown in Nutrient broth at 28°C on a rotary shaker at 150 rpm for 24 h. The cell pellet collected by centrifugation was preserved in RNAlater and sent to Macrogen, Inc. (Seoul, South Korea) for genomic DNA extraction using the DNeasy Blood & Tissue Kit (Qiagen, Hilden, Germany) and whole-genome sequencing. The DNA library was prepared using the TruSeq DNA Nano sample preparation kit (Illumina, San Diego, CA, USA), and then paired-end (2 × 150 bp) sequencing was performed using the Illumina HiSeq XTen platform. Raw reads were trimmed at Q30 using Trimmomatic v0.36 (7). *De novo* assembly was performed using Unicycler v0.5 (8), and the assembly output was assessed using QUAST v5.2 (9). The completeness and contamination of the genome were assessed using BUSCO v5.3.2 (10) and CheckM v1.0.12 (11). Genome annotation was performed independently using the NCBI Prokaryotic Genome Annotation Pipeline (PGAP) v.6.8 (12) and RAST v1.073 (13). The taxonomic classification of the strain was determined using the GTDB-Tk v1.7.0 (14). Secondary metabolite biosynthetic gene clusters (BGCs) were predicted using AntiSMASH v8.0 (15). All software used default parameters unless stated otherwise.

Sequencing yielded 5,020,317 150 bp paired-end reads, with a genome coverage of ~160×. The genome is 4,599,749 bp in size with 56.2% G + C content, 27 contigs, *N*_50_ of 599,098, and *L*_50_ of 2. The assembly’s completeness was 99.1% according to BUSCO and 99.96% according to CheckM, with a contamination level of 0.08%. The strain was identified as *E. roggenkampii*, showing 98.5% ANI with the reference genome GCF_001729805.1. PGAP identified 4,275 protein-coding sequences (CDSs) and 93 RNA genes in the genome, while RAST identified 4,305 CDSs assigned into 371 subsystems. The genome contained genes that enhance plant growth through direct mechanisms like phytohormone production, nutrient acquisition, and siderophore production, as well as indirect mechanisms involving volatile organic compounds (VOCs) synthesis and abiotic stress tolerance (Table 1). Moreover, antiSMASH predicted seven BGCs that may synthesize metabolites that have roles in pathogen control, nutrient uptake, and biofilm formation (16, 17).

**TABLE 1:** Genes associated with direct and indirect mechanisms of plant growth promotion found in the *E. roggenkampii* B1-01 genome

PGP mechanism	Pathway	Associated genes (18)
Phytohormone production	Auxin biosynthesis	*ipdC, trpAB, trpCF*
Nutrient acquisition	Phosphate solubilization	*gcd, phoA, ppx, phnCDE, pstABCS*
	Potassium solubilization	*ackA, mdh, gltA, kdpABC, kefBCFG, kup, trkAH*
	Nitrogen metabolism	*nifJ, nifM, nifS, nifU, narI, nirBD, narGH, nasABDE, glnL, ureABCDEFGH*
Siderophore production	Aerobactin and enterobactin synthesis and transport	*iucC, entF, entD, entS, fepBCG*
Volatile organic compounds (VOCs) production	Acetoin and 2,3-butanediol synthesis	*alsD, butA, ilvBGLHMN*
Stress tolerance	ACC deaminase synthesis	*dcyD*
	Osmolyte production	*otsAB, treBYZ, betAB, ectB, proABC*
	Antioxidant enzyme synthesis	*sod, katEG, gst, gpx, gshAB, ggt, ggpS*
	Heat shock protein synthesis	*grpE, hspQ, htpX, hslR, dnaJK, clpABPX*

## Data Availability

The whole-genome shotgun project of *Enterobacter roggenkampii* B1-01 has been deposited in NCBI GenBank under the accession number JBIQNZ000000000, BioProject PRJNA1171129, and BioSample SAMN44234176. Raw reads were deposited in GenBank under the accession number SRX26331886. Other annotations have also been uploaded to Zenodo.
